# In vivo pressure-flow relation of human cutaneous vessels following prolonged iterative exposures to hypergravity

**DOI:** 10.1152/ajpregu.00010.2023

**Published:** 2023-05-08

**Authors:** Michail E. Keramidas, Roger Kölegård, Håkan Sköldefors, Ola Eiken

**Affiliations:** ^1^Division of Environmental Physiology, Swedish Aerospace Physiology Center, KTH Royal Institute of Technology, Stockholm, Sweden; ^2^Swedish Air Force, Uppsala, Sweden

**Keywords:** gravitoinertial load, G training, limb, myogenic response, skin circulation

## Abstract

The study examined intra- and interlimb variations in cutaneous vessel responsiveness to acute and repeated transmural pressure elevations. In 11 healthy men, red blood cell flux was assessed via laser-Doppler flowmetry on both glabrous and nonglabrous skin regions of an arm (finger and forearm) and leg (toe and lower leg), across a wide range of stepwise increasing distending pressures imposed in the vessels of each limb separately. The pressure-flux cutaneous responses were evaluated before and after 5 wk of intermittent (40 min, 3 sessions per week) exposures to hypergravity (∼2.6–3.3 G; G training). Before and after G training, forearm and lower leg blood flux were relatively stable up to ∼210 and ∼240 mmHg distending pressures, respectively; and then they increased two- to threefold (*P* < 0.001). Finger blood flux dropped promptly (*P* < 0.001), regardless of the G training (*P* = 0.64). At ≤120-mmHg distending pressures, toe blood flux enhanced by ∼40% (*P* ≤ 0.05); the increase was augmented after the G training (*P* = 0.01). At high distending pressures, toe blood flux dropped by ∼70% in both trials (*P* < 0.001). The present results demonstrate that circulatory autoregulation is more pronounced in glabrous skin than in nonglabrous skin, and in nonglabrous sites of the leg than in those of the arm. Repetitive high-sustained gravitoinertial stress does not modify the pressure-flow relationship in the dependent skin vessels of the arm nor in the nonglabrous sites of the lower leg. Yet it may partly inhibit the myogenic responsiveness of the toe’s glabrous skin.

## INTRODUCTION

During arterial pressure perturbations, the cutaneous precapillary vessels exhibit autoregulatory properties enabling adequate tissue perfusion while protecting capillary integrity. Thus, elevations in transmural vascular pressure in the lower body induced either by a transition from recumbent to upright posture or exposure to a high-sustained gravitoinertial force field in the head-to-seat direction (i.e., +Gz; henceforth G denotes +Gz unless otherwise stated) elicit an increase in the tone of dependent skin vessels, through prompt recruitment of the myogenic response and perhaps also of the venoarteriolar reflex, counteracting pressure distension (1–6). Τhe pattern and magnitude of vasoreactivity, however, are not uniform across the cutaneous vascular beds but describe intra- and interlimb heterogeneity. Specifically, circulatory autoregulation is accentuated in glabrous skin regions compared with nonglabrous skin regions (7, 8), as well as in deeper vascular beds compared with superficial vascular beds (9, 10). Furthermore, combining evidence from independent studies, assessing changes in limb heat elimination (an index of cutaneous circulation) by means of a vacuum calorimeter during large increments in local transmural pressure, suggests that the vessels in the lower extremities (toes) are more pressure-resistant than those in the upper extremities (hand; 11–14). Snyder et al. (15) and Wilson et al. (16), however, failed to observe any differences in the skin vasomotion of forearm versus calf during limb dependency and venous congestion, respectively.

It has been presumed that the between-limb variation in the myogenic responsiveness is attributable to the differential pressure loads encountered by each vascular segment regularly across the life span; since in erect posture, the lower limb vasculature is exposed to augmented hydrostatic pressure components (17–20). In support of this notion, we recently provided direct evidence that the in vivo mechanical properties of the cutaneous vessels are indeed plastic, adjusting to the prevailing pressure demands (7). Thus, prolonged repeated intravascular pressure loading induced by 5 wk of intermittent local (arm) exposure to subatmospheric ambient pressure enhanced arteriolar pressure resistance in the nonglabrous (forearm) skin, whereas inhibited, at discrete elevations of the distending pressures, the constrictor responsiveness of the glabrous (finger) skin arterioles. Whether the cutaneous vessels of the legs exhibit similar adaptability to long-term repeated transmural pressure elevations has not been determined directly. Furthermore, if, and to what extent, reoccurring whole body exposures to high G loads, as fighter pilots typically experience while flying high-performance aircraft (21, 22), would be capable of modulating the autoregulatory properties of the dependent skin vessels is unknown. Interestingly, microgravity-related studies have provided inconsistent results with regard to the vasoadaptive capacity of cutaneous vessels to pressure unloading: Wilson et al. (16) found that 14 days of bed rest (a ground-based analog for assessing certain effects of microgravity) attenuated lower leg skin vasoconstriction during venous congestion, whereas Gabrielsen et al. (23) did not observe, after a 20-day bed rest, any changes in the vasomotor function of the foot in response to limb dependency.

Accordingly, this study evaluated the hypothesis that the intrinsic functional responsiveness of cutaneous precapillary vessels to acute local transmural pressure elevations would be more pronounced in the lower limbs than in the upper limbs. We therefore used a within-subject design, wherein blood flux on both glabrous and nonglabrous skin regions of an arm (finger and forearm, respectively) and leg (toe and lower leg, respectively) were assessed continuously via laser-Doppler flowmetry, across a wide range of stepwise increasing distending pressures imposed in the vessels of each limb separately. The study also aimed to investigate whether the pressure-flow cutaneous responses would be influenced by long-term, iterative exposures to hypergravity. On the basis of our previous work (7), we hypothesized that a 5-wk G-training regimen performed in a human-use centrifuge would augment pressure resistance in the nonglabrous skin (i.e., the pressure threshold for distension-induced enhancement of flow would be elevated), but would blunt partly the vasomotor responsiveness of the glabrous skin (i.e., inhibiting the prompt flow reduction occurring at slight-to-moderate distending pressure increments). We anticipated, however, that such training-induced vasoadaptations would be more pronounced in the leg microvasculature, because, during G loading, the precapillary vessels of the lower limb are subjected to markedly higher intravascular pressure increments than those of the arm (24).

## METHODS

### Ethics Approval

The study was approved by the Human Ethics Committee of Stockholm (Ref. No.: 2016/1889-31/4) and conformed to the standards set by the Declaration of Helsinki. Subjects were informed in detail about the experimental procedures before giving their written consent to participate. This work was part of a larger project investigating the effects of hypergravity on the human cardiovascular system (see Ref. 25).

### Subjects

Based on our previous work assessing the effects of 5 wk of intermittent local vascular pressure increments on cutaneous circulation (7), a minimum sample size of eight individuals was determined a priori, using α = 0.05, β = 0.80, and an effect size of 0.75 (G*Power 3.1 software, Heinrich-Heine-Universität, Dusseldorf, Germany; see Ref. 26). Therefore, 11 healthy men [mean (range): age 23 (22–24) years, body mass 80.3 (64.0–104.2) kg, and height 179 (168–190) cm] participated in the study. Subjects were military flight cadets of the Swedish Armed Forces. They, however, were recruited before any flight training and had not been exposed to high G loads within 3 mo before the initiation of the study. All were normotensive, nonsmokers, had no history of any vascular disorder, and were not taking any medication. Also, they were instructed to abstain from alcohol and strenuous exercise for at least 24 h before each trial, to refrain from caffeine during the testing day, and to maintain their sleeping, eating, and exercise routines throughout the study period.

### Study Design

The study was conducted in the experimental facilities (the human-use centrifuge and the hyperbaric chamber) of the Division of Environmental Physiology, Royal Institute of Technology (Solna, Sweden). All subjects attended a testing session before (pretrials) and after (posttrials) a 5-wk G-training regimen. The pretrials were performed 4 days before the first training session and the posttrials 5 days after the last training session. During each testing session, subjects performed, in an alternated order and separated by ∼1 h interval: *1*) an arm pressure-provocation trial and *2*) a lower leg pressure-provocation trial (see *Pressure-provocation trials* for details). For the individual subject, the pre- and posttrials were carried out at the same time of the day, and their sequence remained constant between the testing periods (5 subjects started with the arm-provocation trial and 6 subjects with the leg-provocation trial).

#### Pressure-provocation trials.

**Figure 1.**
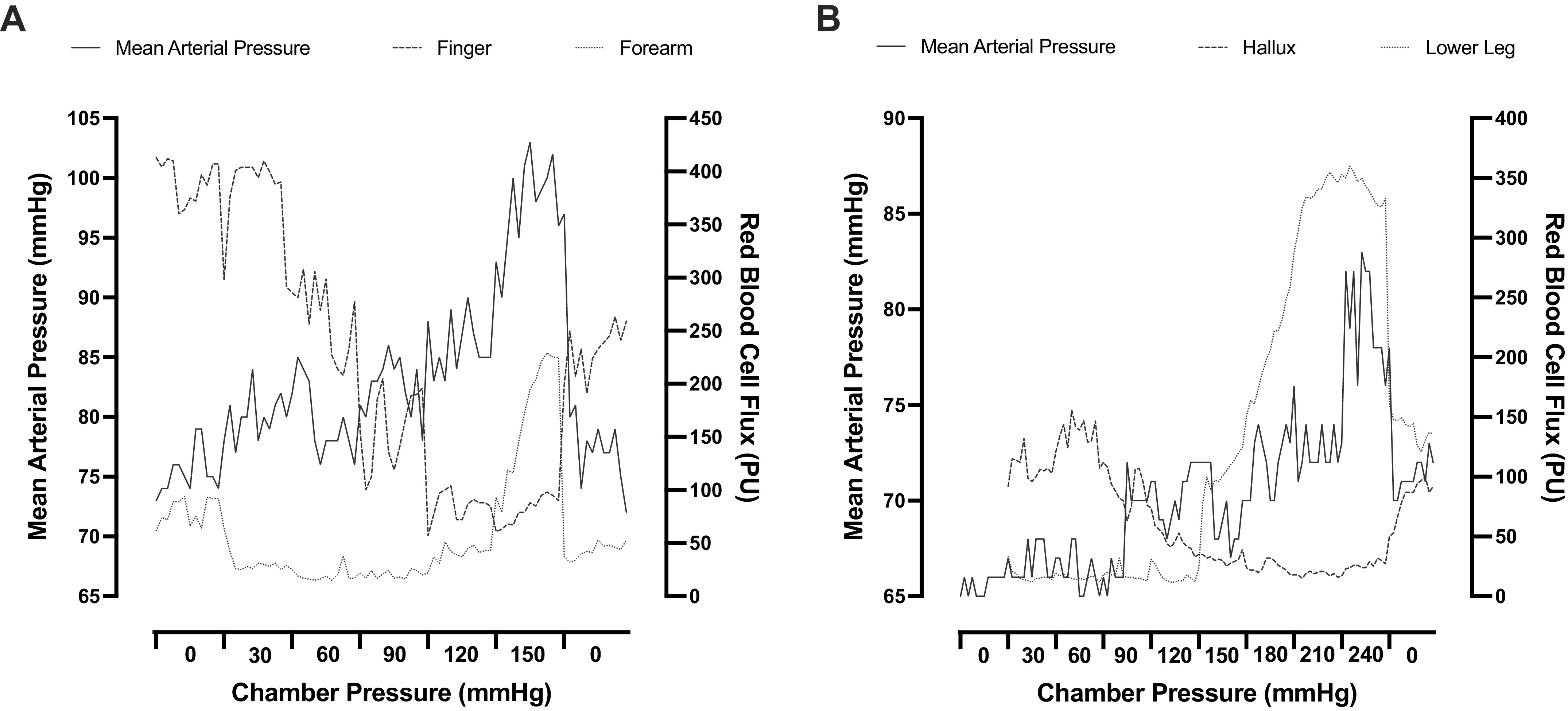
Representative time course recordings of a subject’s mean arterial pressure and cutaneous red blood cell flux obtained during the arm (*A*) and leg (*B*) pressure-provocation trials. PU, perfusion units.

The local intravascular pressures in the vasculature of the left (nondominant) arm and lower leg were increased gradually, using a technique that has been described in detail previously; that is, the subject is confined into a hyperbaric chamber with his/her test arm or lower leg (henceforth, test limb) protruding to the outside of the chamber (27, 28). Specifically, before each trial, subjects, who were dressed in T-shirts and shorts (and in socks in the arm-provocation trial), were accustomed to the laboratory for ∼30 min while being instrumented. They were then seated (in the arm-provocation trial) or laid supine (in the leg-provocation trial) in a hyperbaric chamber with the test limb extended through an opening in the chamber door. The test limb was hermetically sealed to the opening slightly distally of the axilla or proximally of the knee, respectively, using a short self-sealing rubber sleeve and was supported at the level of the heart by a stand placed outside of the chamber. After a 10-min resting period, each trial commenced with a 3-min baseline phase, during which the pressure in the chamber was atmospheric. Thereafter, the chamber pressure was increased by 30 mmHg every 3 min, up to +150 mmHg in the arm-provocation trial and up to +240 mmHg in the leg-provocation trial. When the chamber pressure is elevated, the pressure increases in all tissues enclosed in the chamber and hence transmitted almost undistorted to the precapillary vessels of the test limb; eventually, venous pressure in the test limb will increase until it overcomes pressure in the veins enclosed in the chamber and blood flow through the limb reassumes within seconds (see Fig. 1; 27, 29, 30). After the last pressure plateau, the chamber pressure was rapidly released. During all trials, the ambient temperature at the site of the test limb was maintained at ∼25°C.

#### G-training regimen.

Subjects undertook three 40-min G-training sessions per week during a 5-wk period, in a 7.25-m radius human-use centrifuge (ASEA, Sweden). During each session, the subjects, who were dressed in T-shirts, long trousers, socks, and shoes, sat in the tangentially pivoted centrifuge gondola, wherein the seat back reclines 28° from the vertical. Throughout the session, subjects maintained their arms on their laps; thus, the hands and forearms were at a vertical level 15–25 cm below the heart. Subjects were requested to remain relaxed while the G load was oscillated, at 1-min intervals and at 0.5 G·sec^−1^ transition rate, between idle speed (1.4 G) and a G load corresponding to ∼85% of the individual-relaxed G tolerance. The goal was to maintain the training stimulus (G load) as high as possible without inducing adverse events in terms of G-induced loss of consciousness (G LOC), almost loss of consciousness (A LOC), or loss of foveal vision (black out). Thus, throughout the training regimen, the G load was individually adjusted so that the second step of each high G load period was slightly (0.2–0.4 G) below the level inducing loss of peripheral vision (for more details on the training protocol, see Ref. 25). The mean (range) G load imposed was 2.8 (2.6–3.3) G in *week 1*, 3.0 (2.6–3.6) G in *week 2*, 3.1 (2.6–3.6) G in *week 3*, 3.1 (2.8–4.0) G in *week 4*, and 3.3 (2.9–4.1) G in *week 5*. The G load was measured by means of an analog accelerometer mounted in front of the subjects at a vertical level approximately corresponding to that of their heart. The centrifuge was controlled by an “open-loop” system using preset G-time profiles. During all sessions, the air temperature in the gondola was maintained within the range of 22°C–26°C.

### Measurements

In this study, only data from the pressure-provocation trials are presented. Heart rate (HR) was derived from electrocardiographic recordings using a bipolar precordial lead (Physiocontrol Lifepak 8; Physio-Control Corp., Redmond, WA). Systolic (SAP), diastolic (DAP), and mean (MAP) arterial pressures were measured using a volume-clamp technique (Portapres; TNO, Amsterdam, The Netherlands), with the pressure cuff placed around the middle phalanx of the third finger inside the chamber and with the reference pressure transducer positioned at the level of the heart. The Portapres-derived values were verified, at approximately *minute 2* of each pressure plateau, by sphygmomanometry (Riva-Rocci technique) performed, on the brachial artery of the same arm, always by the same physician who accompanied the subjects inside the chamber throughout the trial. HR and arterial pressures were recorded at a frequency of 200 Hz with a data acquisition system (Biopac System, Inc., Santa Barbara, CA) connected to a computer, which was placed outside the chamber. During the baseline period, and during the last 30 s of each pressure plateau, subjects were asked to rate the test-limb pain using a 10-point scale (from 0, no pain, to 10, maximal pain).

Local skin red blood cell flux was monitored at a rate of 40 Hz by laser-Doppler flowmetry (VMS-LDF2; Moor Instruments, Axminster, UK). Τhe optic probe (VP7b; Moor Instruments, UK) was positioned in a local heater (VHP1; Moor Instruments, UK) and was affixed with double-sided adhesive tape to the skin on *1*) the dorsal side of the forearm (nonglabrous skin) and the palmar side of the distal phalanx of the index finger (glabrous skin) of the test arm during the arm-provocation trial and *2*) the lateral side of the lower leg (i.e., over the midportion of the tibialis anterior muscle; nonglabrous skin) and the plantar side of the distal phalanx of the hallux (glabrous skin) of the test leg during the leg-provocation trial. To ensure similar surface temperatures across the anatomical regions of interest, as well as the provocation trials and the subjects, the skin temperature of the scanned sites was clamped at 34°C throughout (31). The probes were always placed by the same investigator, who employed anatomical measurements to secure that, in all trials, they were positioned approximately at the same spots. Data were reported as absolute values of red blood cell flux [in perfusion units (PU)] and cutaneous vascular conductance (CVC; calculated as cutaneous red blood cell flux divided by MAP, in PU·mmHg^−1^). Since the laser-Doppler values were not normalized to a maximally induced value (e.g., via local heating of the scanned skin area), our observations were focused on potential interregional and intertrial variations on the response pattern.

### Statistical Analyses

Data from the baseline and each pressure plateau were calculated and presented as averages of the final 2 min period. Skin red blood cell flux and CVC were plotted as functions of arterial distending pressure, which was approximated by adding chamber pressure to MAP (7). Normality of distribution for all datasets was assessed using the Shapiro–Wilk test. A two-way [testing period (pre- × posttrial) × distending or supra-atmospheric pressure] repeated-measures analysis of variance (ANOVA) was used for all physiological variables. Mauchly’s test was conducted to assess the sphericity and, if necessary, the Greenhouse–Geiser *ɛ* correction was used to adjust the degrees of freedom. When ANOVA revealed significant effects, multiple pairwise comparisons were performed with Tukey’s honestly significant difference post hoc test. Differences in the perceived pain were evaluated with Friedman’s test, followed by a Wilcoxon test. Kendall’s coefficient of concordance (*W*) was also estimated to assess the internal consistency of pain ratings. Statistical analyses were conducted using STATISTICA 8.0 (StatSoft, Inc., Tulsa, OK), and figures were produced using Prism 9.3 (GraphPad Software, Inc., San Diego, CA). Unless otherwise stated, data are presented as mean values with 95% confidence intervals [CIs], which were calculated using a noncentral *t* distribution. The α level of significance was set a priori at 0.05.

## RESULTS

### Arm Pressure-Provocation Trial

During both trials, SAP, DAP, and MAP increased gradually (*P* < 0.001), whereas HR remained unaltered (*P* = 0.41; Table 1). During the baseline phase, and at slight and moderate chamber pressure elevations, arterial pressures (*P* ≤ 0.05) and HR (*P* = 0.03) were lower after the G training. When the chamber pressure was increased by ≥60 mmHg, subjects perceived pain in the test arm (*P* < 0.001; Kendall’s *W* = 0.90); the pain sensation was not affected by the G training (*P* = 0.30; Table 1).

**Table 1.** Systolic, diastolic, and mean arterial pressures, heart rate, and perceived local pain obtained over each chamber pressure plateau during the arm vascular pressure-provocation trial, performed before and after a 5-wk G-training regimen

	Pre-G Training						Post-G Training					
	Baseline	30 mmHg	60 mmHg	90 mmHg	120 mmHg	150 mmHg	Baseline	30 mmHg	60 mmHg	90 mmHg	120 mmHg	150 mmHg
SAP, mmHg	120 [5]	123 [6]	128 [7]	132 [7]^a^	136 [8]^a^	144 [10]^a^	108 [7]*	112 [7]*	114 [8]*	120 [7]^a^*	129 [6]^a^*	141 [8]^a^
DAP, mmHg	64 [7]	66 [5]	69 [8]	69 [6]	72 [7]^a^	76 [9]^a^	58 [7]	57 [5]*	61 [7]*	67 [8]^a^	70 [6]^a^	81 [7]^a^
MAP, mmHg	83 [6]	85 [5]	89 [7]	90 [6]^a^	94 [6]^a^	99 [6]^a^	75 [6]*	75 [5]*	78 [6]*	85 [6]^a^	90 [6]^a^	101 [7]^a^
HR, beats/min	71 [8]	69 [9]	72 [7]	68 [7]	71 [6]	69 [7]	64 [6]*	63 [8]	65 [6]*	69 [5]	67 [7]	70 [4]
Pain, 0–10	0	0 (0–1)	1 (0–3)^a^	2 (1–4)^a^	4 (1–7)^a^	5 (3–9)^a^	0	0 (0–1)	1 (0–3)^a^	3 (1–4)^a^	4 (1–5)^a^	5 (3–8)^a^

Values are represented as mean [95% confidence interval] for systolic (SAP), diastolic (DAP), and mean arterial pressures (MAP) and heart rate (HR). Values are represented as mean (range) for the pain ratings. *n* = 11 men. SAP, DAP, MAP, and HR were analyzed with a two-way repeated-measures ANOVA, followed by Tukey’s honestly significant difference post hoc test, and the pain ratings were analyzed with Friedman’s test, followed by a Wilcoxon test. Significantly different ^a^from baseline and *from the pre-G-training trial.

**Figure 2.**
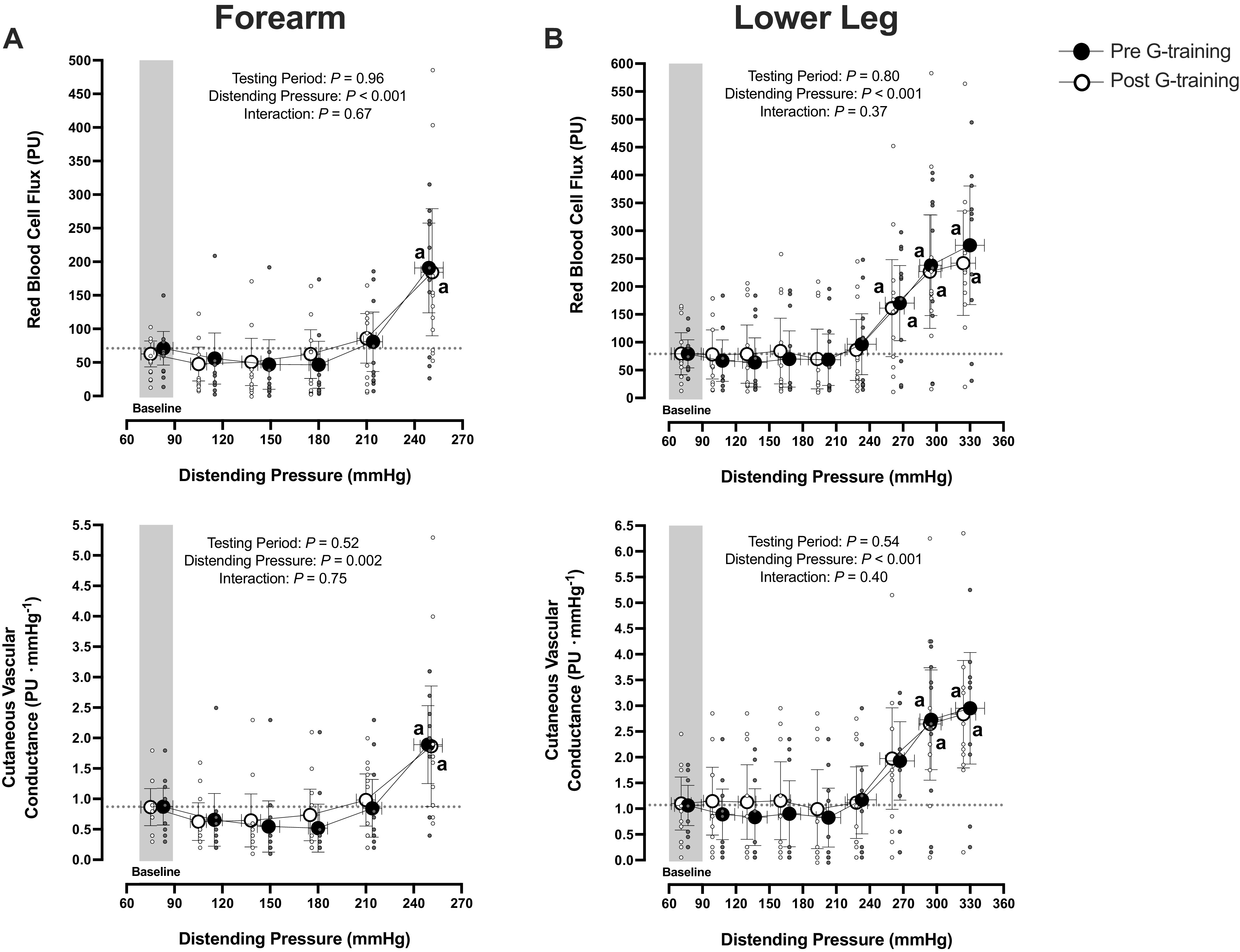
Forearm (*A*) and lower leg (*B*) cutaneous red blood cell flux (*top*) and vascular conductance (*bottom*) of the test limb during the local vascular pressure-provocation trials, performed before and after a 5-wk G-training regimen (*n* = 11 men). Values are represented as mean [95% confidence interval]. Data were analyzed with a two-way repeated-measures ANOVA, followed by Tukey’s honestly significant difference post hoc test. ^a^Significantly different from baseline. PU, perfusion units.

**Figure 3.**
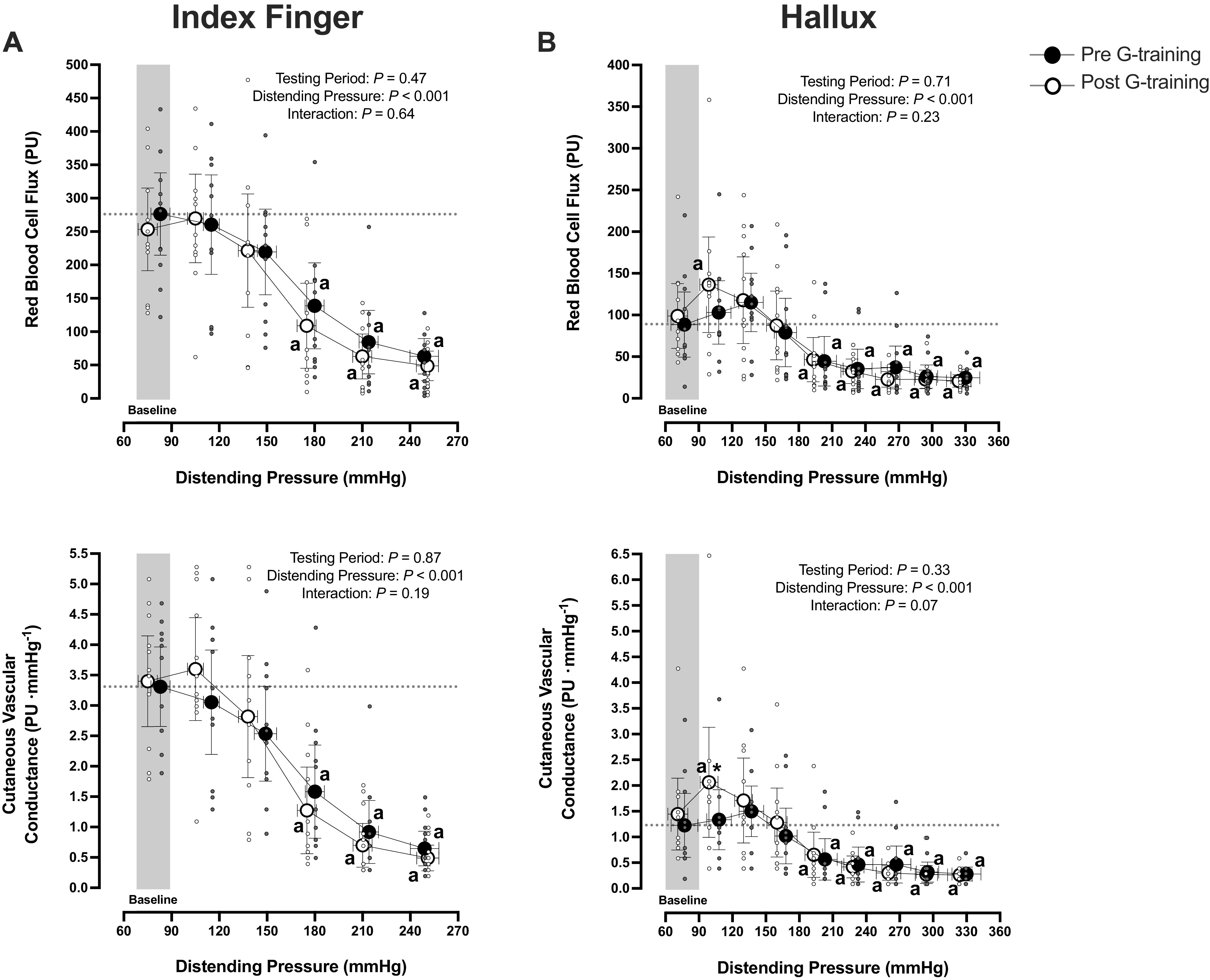
Index finger (*A*) and hallux (*B*) cutaneous red blood cell flux (*top*) and vascular conductance (*bottom*) of the test limb during the local vascular pressure-provocation trials, performed before and after a 5-wk G-training regimen (*n* = 11 men, except for at peak distending pressures of the pretrial where *n* = 10). Values are represented as mean [95% confidence interval]. Data were analyzed with a two-way repeated-measures ANOVA, followed by Tukey’s honestly significant difference post hoc test. Significantly different ^a^from baseline and *from the pre-G-training trial. PU, perfusion units.

Forearm skin blood flux and CVC were relatively stable at slight and moderate elevations of distending pressures (Fig. 2*A*). At the peak (i.e., >210 mmHg) distending pressures of both trials (*P* > 0.05), however, they increased approximately two- to threefold (*P* < 0.001). Finger skin blood flux and CVC dropped promptly (*P* < 0.001) and in a similar manner in both trials (flux: *P* = 0.64, CVC: *P* = 0.19; Fig. 3*A*).

### Leg Pressure-Provocation Trial

Before the G training, one trial was terminated immediately after the completion of the 210-mmHg plateau, because the subject complained of severe pain in the test leg.

During both trials, SAP, DAP, and MAP increased gradually (*P* < 0.001), whereas HR remained relatively stable (*P* = 0.10; Table 2). During the baseline phase, and at slight and moderate chamber pressure elevations, the arterial pressures were lower after the G training (*P* ≤ 0.01). When the chamber pressure was increased by ≥90 mmHg, subjects perceived pain in the test leg (*P* < 0.001; Kendall’s *W* = 0.87); the pain sensation was not affected by the G training (*P* = 0.20; Table 2).

**Table 2.** Systolic, diastolic, and mean arterial pressures, heart rate, and perceived local pain obtained over each chamber pressure plateau during the lower leg vascular pressure-provocation trial, performed before and after a 5-wk G-training regimen

	Pre-G Training									Post-G Training								
	Baseline	30 mmHg	60 mmHg	90 mmHg	120 mmHg	150 mmHg	180 mmHg	210 mmHg	240 mmHg	Baseline	30 mmHg	60 mmHg	90 mmHg	120 mmHg	150 mmHg	180 mmHg	210 mmHg	240 mmHg
SAP, mmHg	118 [12]	119 [11]	119 [12]	119 [11]	127 [11]	128 [12]	131 [11]^a^	128 [12]	133 [13]^a^	108 [7]*	105 [10]*	105 [9]*	106 [10]*	110 [9]*	116 [11]*	119 [12]*	122 [10]^a^	125 [12]^a^
DAP, mmHg	57 [7]	58 [8]	57 [7]	58 [7]	61 [8]	61 [9]	66 [10]^a^	64 [6]	69 [8]^a^	53 [6]	51 [4]*	53 [5]	53 [5]	55 [5]	58 [5]	61 [5]^a^	64 [6]^a^	63 [6]^a^
MAP, mmHg	77 [8]	78 [7]	77 [7]	78 [7]	83 [7]	83 [9]	87 [9]^a^	85 [7]	90 [9]^a^	71 [6]	69 [5]*	70 [5]*	70 [6]*	73 [6]*	78 [6]	80 [7]^a^	84 [7]^a^	84 [7]^a^
HR, beats/min	65 [5]	68 [5]	65 [5]	63 [5]	65 [5]	65 [4]	67 [5]	66 [5]	68 [7]	64 [5]	60 [5]	60 [6]	60 [6]	61 [6]	63 [5]	63 [6]	67 [6]	66 [7]
Pain, 0–10	0	0	0 (0–1)	1 (0–3)^a^	2 (0–5)^a^	3 (1–6)^a^	4 (1–7)^a^	4 (1–9)^a^	5 (2–10)^a^	0	0 (0–1)	0 (0–1)	1 (1–2)^a^	2 (1–3)^a^	2 (1–5)^a^	3 (1–5)^a^	3 (2–6)^a^	4 (2–7)^a^

Values are represented as mean [95% confidence interval] for systolic (SAP), diastolic (DAP), and mean arterial pressures (MAP) and heart rate (HR). Values are represented as mean (range) for the pain ratings. *n* = 11 men, but at peak distending pressures in the pretrial *n* = 10. SAP, DAP, MAP, and HR were analyzed with a two-way repeated-measures ANOVA, followed by Tukey’s honestly significant difference post hoc test, and the pain ratings were analyzed with Friedman’s test, followed by a Wilcoxon test. Significantly different ^a^from baseline and *from the pre-G-training trial.

In both trials, lower leg skin blood flux and CVC remained virtually unchanged up to ∼240-mmHg distending pressure, and then they increased two- to threefold (*P* ≤ 0.03; Fig. 2*B*). At the peak distending pressures, the increase in lower leg blood flux did not differ between trials (*P* = 0.97). Upon the initiation of both trials, hallux skin blood flux and CVC increased rapidly, especially after the G training (flux: *P* = 0.05, CVC: *P* = 0.01; Fig. 3*B*). Thereafter (≤120 mmHg), hallux blood flux and CVC dropped approximately three- to fourfold (*P* < 0.001), with no intertrial differences (*P* > 0.05).

## DISCUSSION

The study examined, in healthy young men, regional variations in cutaneous vessel responsiveness to acute and repeated transmural pressure elevations. The main findings were that *1*) the pressure-flow responses are more pronounced in glabrous skin than in nonglabrous skin, both in the upper and lower limbs; *2*) cutaneous vessels in nonglabrous areas of the leg are more pressure-resistant than those of the arm; and *3*) prolonged increases in hydrostatic pressure gradients induced by 5 wk of repetitive high-sustained gravitoinertial stress (G training) do not modify the autoregulatory capacity of the skin vessels of the arm or of the nonglabrous sites of the leg, but may partly inhibit the constrictor responsiveness of the lower limb’s glabrous skin at discrete increments (≤+60 mmHg) in distending pressure.

### Pressure-Flow Responses in Nonglabrous Skin

At slight to moderate elevations of the distending pressure, blood flux in the nonglabrous skin of both limbs remained virtually unaltered, presumably through rapid recruitment of the myogenic reflex (1, 32, 33) and possibly also through the venoarteriolar response (2, 3, 5); whereas at high distending pressures, it increased two- to threefold. Still, and in favor of the previous premise (11–14), the lower leg skin vessels were capable of withstanding markedly higher intravascular pressure loads than the forearm skin vessels: the distending pressure threshold for enhancing flow was at ∼210 mmHg for the forearm and at ∼240 mmHg for the calf (*P* = 0.01). The overall pattern of response, as well as the interlimb differences in the nonglabrous skin distensibility, did resemble those typically observed in the skeletal muscle arteries and arterioles of the arm versus lower leg during stepwise increments in local transmural pressure (17, 25). Corroboratively, Lott et al. (19) have shown that the myogenic response to acute elevations in transmural pressure was greater in vascular beds perfused by the femoral artery than those perfused by the brachial artery. Hodges and Del Pozzi (34) have also found, by means of spectral analysis, that, in basal thermoneutral conditions, the myogenic, endothelial, and sympathetic activity are higher in the leg than in the arm microvessels. The enhanced stiffness of the lower leg skin vessels can probably be ascribed not only to augmented myogenic tone but also to structural adaptations ensuing from the habitual repeated hydrostatic pressure loading while in erect posture during normal ambulatory life. It may thus reflect limb-specific differences in the passive elastic recoil properties of the cutaneous vessel wall, associated with variations in wall thickness, wall thickness-to-lumen ratio, and/or in the concentration and arrangement of collagen and elastin (35–37). Of interest in this regard is that, in giraffes, in which the lower extremities are subjected chronically to exaggerated hydrostatic pressures, the mechanical resilience of the leg skin vessels resides primarily on the high collagen density (38, 39), and not on any media hypertrophy; at variance with the differences noted between the carotid and tibial arteries (40), the thickness as well as the wall-to-lumen ratio in the skin arterioles of the leg and neck are similar (38, 41). Furthermore, some (20, 42), albeit not all (43), human-based studies have described between-limb variability in the vascular responsiveness to adrenergic stimulation, with the lower limbs exhibiting an augmented vasoconstrictor sensitivity.

**Figure 4.**
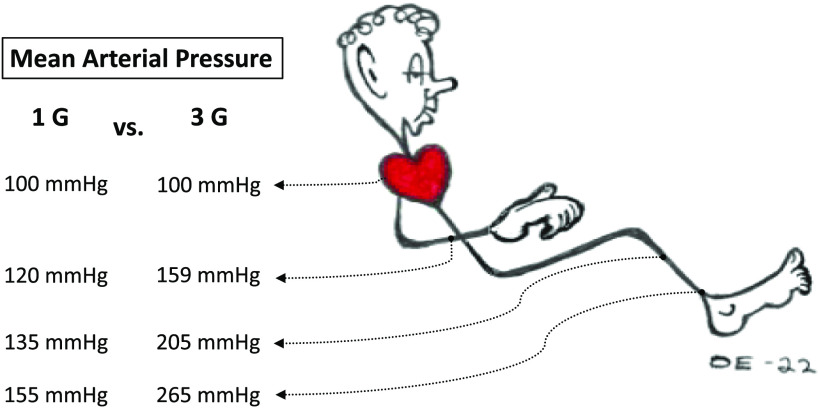
Schematic illustration of the subject’s position in the centrifuge gondola during the G-training sessions, and the approximated mean arterial pressure gradients encountered at 1 and 3 G. Adapted from Eiken et al. (25).

Recently, we yielded direct evidence that the mechanical properties of nonglabrous skin vessels are plastic, adjusting to the prevailing pressure demands in much the same way as those of deeper precapillary vessels. Namely, the pressure-flow responses of the forearm skin vessels (7) and the vascular circuits perfused by the brachial artery (44) alike were blunted after 5 wk of intermittent exposure of an arm to elevated transmural vascular pressures. Based on this finding, we therefore anticipated that transient increments in hydrostatic pressure gradients invoked by 5 wk of iterative exposures to sustained high G forces (∼2.6–3.3 G) would, in a similar manner, enhance the stiffness of leg arterioles, regardless of whether these are located in the nonglabrous regions of the skin or in the skeletal muscles. Indeed, the repetitive gravitoinertial stress reduced the pressure-flow and pressure-diameter increments at ankle level in the posterior tibial artery (25), whereas, contrary to our hypothesis, it failed to induce any vasoadaptive modifications in the cutaneous vessels of the lower leg. The conflicting results between the present and the previous (7, 25) study interventions may be explained by differences in the training-stimulus features. Thus, in our previous pressure training study, the intravascular pressure increments obtained during the training sessions were at or somewhat above the pretraining distending pressure thresholds both the arm skin vessels (7) and of the deeper arm arteries/arterioles (44). During the present G-training sessions, however, the precapillary pressures at the forearm and hand that were maintained slightly below the heart level were approximated to the range, on average, between 135 and 170 mmHg (Fig. 4), which were considerably lower than the distending pressure thresholds of the forearm (i.e., ∼210 mmHg; Fig. 2*A*). Evidently, such a stimulus intensity was inadequate to trigger any training-related circulatory alterations in the arm vasculature. Likewise, at the site of the lower leg skin blood flux measurement, the precapillary distending pressure was estimated to vary from 200 to 240 mmHg during the training sessions (Fig. 4), which was below or approaching the pretraining distension threshold (i.e., ∼240 mmHg; Fig. 2*B*). By contrast, in the tibial artery, at the level of the ankle ∼20–25 cm below the lower leg skin site, the local intraarterial pressure during the G-training sessions varied between 245 and 280 mmHg (Fig. 4), and hence repeatedly and substantially exceeded the pretraining pressure thresholds for arterial (269 mmHg) and arteriolar (261 mmHg) distensions, respectively (25). In addition, it cannot be ruled out that the time course of physiological adjustments occurring in different anatomical compartments of the same limb varies with the muscle vessels being more responsive than the skin vessels.

### Pressure-Flow Responses in Glabrous Skin

Contrary to in nonglabrous skin, and in line with previous observations (1, 7, 8), the palmar/plantar side of the digits displayed augmented pressure-flow responses. Thus, in both limbs alike, acral blood flux dropped substantially, when the distending pressures exceeded ∼180 mmHg, and it almost plateaued thereafter (Fig. 3). However, at discrete (i.e., ≤120 mmHg) distending pressures, a between-limb heterogeneity was noted: finger blood flux remained unaltered, or even slightly decreased, whereas hallux blood flux appeared to increase transiently. Considering that the enhanced pressure-flow response of the glabrous skin is governed by the high presence of arteriovenous anastomoses (AVAs; 7, 8, 45), and presumably is determined by the AVAs’ constrictor sensitivity to sensory (e.g., noxious) stimuli (46–48), we speculate that the chronic reappearance of elevated transmural pressures in the feet induced by orthostasis may have desensitized the reflex function of AVAs in the toe at discrete increments in the distending pressure. The existence of such desensitization seems to be supported by the finding that the initial increase in hallux blood flux was more prominent after the G training. Likewise, the pressure-induced reduction in finger blood flux was inhibited partly after 5 wk of local pressure training (7). The G-training regimen, nevertheless, did not modify the reactive response of glabrous skin at moderate and high distending pressures. Finally, by the current experimental design, we are unable to illuminate whether the glabrous skin of the leg is more pressure-resistant than that of the arm, given that, in both limbs, blood flux remained minimal at the highest distending pressures, and the distending pressure thresholds for enhancing flow were hence not attained.

### Methodological Considerations

It is well known that the G-induced augmentation of intravascular hydrostatic pressure gradients may lead to the development of petechial hemorrhages (aka “high-G measles”) in the dependent regions of the body (especially on the foot, leg, buttocks, and forearm), typically lasting for 24–48 h after the G exposure (49). Anecdotal evidence from military fighter pilots suggests that the incidence and severity of petechiasis is aggravated during the initial G exposures performed, for instance, after a prolonged layoff period, whereas their occurrence seems to diminish gradually over successive G exposures. Notably, in the present study, we nor the subjects observed, at any stage of the G training, such petechia, which might have confounded the skin blood flux measures.

In general, blood vessel distensibility may be affected by the degree of counter pressure generated by the surrounding tissues (17). Yet the higher stiffness in the calf than the forearm vessels cannot be ascribed to difference in tissue pressure, not only because the investigated vascular beds were located outside the fascial compartments of the limb but also because, based on the impedance-plethysmography measurements (these data have been presented in details in our previous work; see Ref. 25), the pressure-evoked tissue volume expansion was more pronounced in the forearm than in the lower leg, as well as in the pre- than in the post-G-training leg pressure-provocation trials.

We used laser-Doppler flowmetry to assess skin blood flux, which constitutes a qualitative, rather than a quantitative, measure of perfusion (50). The technique, commonly used to evaluate vasomotion in glabrous skin regions abundant in AVAs (45), may though predominantly reflect capillary perfusion and not to the same extent blood passing via the AVAs, which are located deeper in the skin (51). For instance, Eriksen and Lossius (52) failed to detect some of the high velocities in AVAs, while using a laser light wavelength of 632.8 nm and a low-pass filter of 12 kHz. The same authors, however, were able to record flow through AVAs, when they used a longer wavelength of emitted light (820 nm), enhancing the depth of skin penetration, and a higher low-pass filter (21 kHz), allowing the detection of a greater range of blood velocities (53). In this study, the noise-limiting filter was set at 15 kHz and the emitted wavelength was 780 nm. We thus cannot rule out that, during the pressure-provocation trials, the AVAs were forced open, leading to an undetected shunting of blood directly from small arteries to small veins. Regardless, even if the AVAs were open at markedly high distending pressures, flow in the precapillary resistance vessels of limbs’ glabrous skin failed to increase.

It might also be argued that elevations in the chamber temperature associated with the pressure increments (i.e., because of the “Guy-Lussac’s law”) may have enhanced the subjects’ whole body thermal state, thereby confounding the skin blood flux recordings on the test limb. Previous works, however, have indicated that a marked thermal strain is required to attenuate the venoarteriolar constrictor response; namely, by means of direct local heating of skin to >38°C (31) or increases in internal temperature by >0.5°C (54, 55). Although no measurements of skin or core temperature were performed in this study, it is highly unlikely that subjects’ thermal state was affected markedly during the provocation trials. Thus, theoretically, during the courses of the arm and leg pressure distension trials, chamber gas temperature could have increased to 28°C or 29°C and 31°C or 32°C at the final pressure step of the respective trial. It can, however, be presumed that in practice, the temperature elevations were considerably less, due to the concomitant gas cooling effect exerted by the chamber walls and interior. In addition, the ambient temperature surrounding the scanned skin sites was always maintained at 34°C, eliminating any effect of local temperature variations.

### Perspectives and Significance

In conclusion, present findings further confirm the spatial heterogeneity in the intrinsic property of human limb cutaneous vessels to oppose local pressure perturbations. Thus, circulatory autoregulation is more robust in the glabrous skin regions than in the nonglabrous skin regions, as well as in the nonglabrous areas of the leg than in those of the arm microvasculature. In addition, prolonged repeated augmentations of the intravascular hydrostatic pressure gradients provoked by 5 wk of intermittent exposures to hypergravity (∼2.6–3.3 G) do not appear to modify the pressure-flow relationship in the dependent skin vessels of the arm nor in the nonglabrous sites of the lower leg. Yet repetitive high-sustained gravitoinertial stress may temporarily inhibit the circulatory responsiveness of the toe’s glabrous skin.

## DATA AVAILABILITY

Data will be made available upon reasonable request.

## GRANTS

The study was funded by the Swedish Armed Forces (Grant No. 9220905) (to M.E.K., R.K., and O.E.); M.E.K. was supported by a salary grant from the Kungliga Tekniska Högskolan (KTH)-Royal Institute of Technology (Grant No. C-2020-0748).

## DISCLOSURES

No conflicts of interest, financial or otherwise, are declared by the authors.

## AUTHOR CONTRIBUTIONS

M.E.K., R.K., and O.E. conceived and designed research; M.E.K., R.K., H.S., and O.E. performed experiments; M.E.K. and R.K. analyzed data; M.E.K. and O.E. interpreted results of experiments; M.E.K. and O.E. prepared figures; M.E.K. drafted manuscript; M.E.K. and O.E. edited and revised manuscript; M.E.K., R.K., H.S., and O.E. approved final version of manuscript.
